# Transporter engineering for improved tolerance against alkane biofuels in *Saccharomyces cerevisiae*

**DOI:** 10.1186/1754-6834-6-21

**Published:** 2013-02-13

**Authors:** Binbin Chen, Hua Ling, Matthew Wook Chang

**Affiliations:** 1 Division of Chemical and Biomolecular Engineering, School of Chemical and Biomedical Engineering, Nanyang Technological University, 62 Nanyang Drive, Nanyang 637459, Singapore

**Keywords:** Biofuels, ABC transporters, *S. cerevisiae*, Alkanes, Tolerance

## Abstract

**Background:**

Hydrocarbon alkanes, components of major fossil fuels, are considered as next-generation biofuels because their biological production has recently been shown to be possible. However, high-yield alkane production requires robust host cells that are tolerant against alkanes, which exhibit cytotoxicity. In this study, we aimed to improve alkane tolerance in *Saccharomyces cerevisiae*, a key industrial microbial host, by harnessing heterologous transporters that potentially pump out alkanes.

**Results:**

To this end, we attempted to exploit ABC transporters in *Yarrowia lipolytica* based on the observation that it utilizes alkanes as a carbon source. We confirmed the increased transcription of ABC2 and ABC3 transporters upon exposure to a range of alkanes in *Y. lipolytica*. We then showed that the heterologous expression of ABC2 and ABC3 transporters significantly increased tolerance against decane and undecane in *S. cerevisiae* through maintaining lower intracellular alkane level. In particular, ABC2 transporter increased the tolerance limit of *S. cerevisiae* about 80-fold against decane. Furthermore, through site-directed mutagenesis for glutamate (E988 for ABC2, and E989 for ABC3) and histidine (H1020 for ABC2, and H1021 for ABC3), we provided the evidence that glutamate was essential for the activity of ABC2 and ABC3 transporters, with ATP most likely to be hydrolyzed by a catalytic carboxylate mechanism.

**Conclusions:**

Here, we demonstrated that transporter engineering through expression of heterologous efflux pumps led to significantly improved tolerance against alkane biofuels in *S. cerevisiae*. We believe that our results laid the groundwork for developing robust alkane-producing yeast cells through transporter engineering, which will greatly aid in next-generation alkane biofuel production and recovery.

## Background

The development of renewable biofuels such as bio-ethanol [1], butanol [2], bio-diesel [3-5] and jetfuels [6] helps to address energy security and climate change concerns. Recently, the biological production of hydrocarbon alkanes, components of major fuels, has drawn much attention because alkanes have high energy content and are compatible with existing transportation infrastructure. In addition, alkanes have been widely used as an organic solvent in biochemical processes in an effort to improve substrate solubility, enzyme stability and specificity [7,8]. In nature, alkanes are found to be produced from fatty acid metabolites in microorganisms [9,10], insects [11] and plants [12]. Recently, an alkane biosynthetic pathway was identified and characterized in cyanobacteria and re-constructed in *Escherichia coli*[10,13]. In addition, plant alkane biosynthesis pathway was reconstituted in yeast [14].

Despite this promising potential of microbial alkane production, yields and titers are key consideration for industrial-scale production. Further, alkane biofuel production can potentially be affected by the product toxicity as alkanes are proven to be toxic to microorganisms [15,16]. The toxicity of products can be evaluated based on log *P*_*ow*_ which represents the logarithm of partition coefficients in n-octanol and water [17]. Organic products with a log *P*_*ow*_ between 1.5 and 6.0 are extremely toxic for microorganisms and other living cells, such as nonane (log*P*_OW_ = 5.5) and decane (log*P*_OW_ = 6.0) [18]. These alkane products interact preferentially with cytoplasmic membrane, therefore disorganizing its structural integrity. Disruption of membrane structure impairs vital functions, such as the loss of ions, metabolites, lipids, and proteins, and the dissipation of the pH gradient and electrical potential. In addition, several studies have shown that tolerance improvement can lead to clear increases in biofuel production [19-21]. Consequently, there is an urgent need to develop robust microbial cell factories that are tolerant to alkane biofuels.

To overcome biofuel toxicity, several engineering strategies have been designed. Alper et al. [19] employed a global transcription machinery engineering (gTME) approach to improve ethanol tolerance. Stanley et al. [22] used an adaptive evolution engineering method to select stable ethanol-tolerant mutants of *S. cerevisiae*. Hou et al. [23] developed novel genome shuffling method to improve biofuel tolerance, whereas Kang et al. [24] improved microbial tolerance to isooctane through identification and reconstitution of genetic regulatory networks. However, these classical tolerance-improving strategies can be time consuming and laborious. Recently, efflux pumps were utilized to alleviate product toxicity and increase final productivity because of their capability to pump out target products from cells [21,25]. However, no attempts have been made to exploit efflux pumps for alkane biofuel transport in *S. cerevisiae,* a key biofuel cell factory. Hence, in this study, we focused on identifying efflux pumps that potentially transport alkane biofuels and harnessing those pumps as a direct mechanism for increasing tolerance through efflux pumping of alkanes from cells.

To this end, we considered ATP-binding cassette (ABC) transporters of *Yarrowia lipolytica*, an oleaginous yeast that efficiently assimilates and utilizes hydrophobic substrates such as alkanes, fatty acids and lipids [26-28]. Further, the mutants lacking part of ABC transporters show defective phenotypes for alkane utilization as a carbon source [29,30]. Consequently, in this study, we attempted to improve the tolerance of *S. cerevisiae* against alkanes using *Y. lipolytica* ABC transporters. Notably, we demonstrated that ABC2 and ABC3 transporters maintained 5 and 30-fold lower intracellular decane and undecane levels respectively, and significantly improved tolerance in *S. cerevisiae*, evidenced by about 80-fold increase in the tolerance limit against decane.

## Results

### Transcription activity of ABC transporters with alkanes

**Figure 1:**
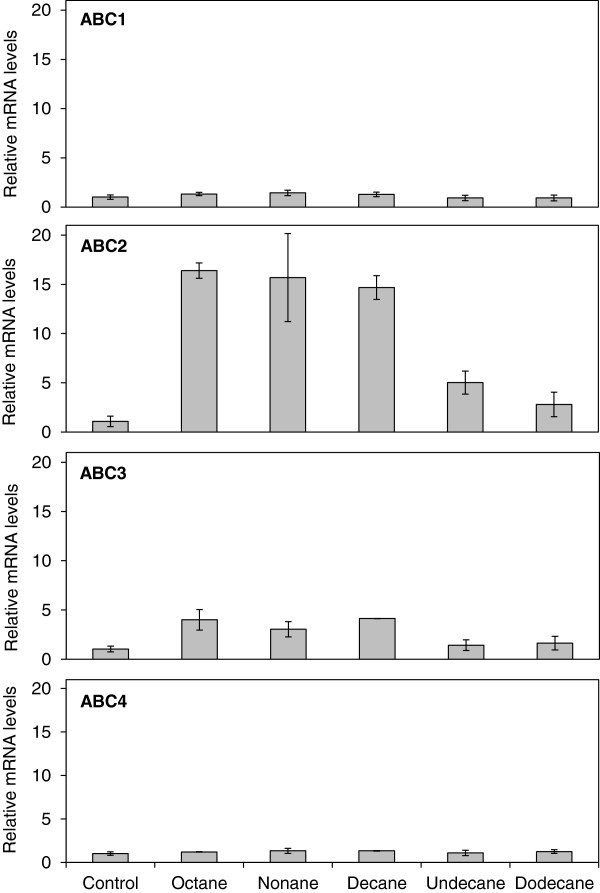
**mRNA transcript levels of *ABC1–4 *genes in *Y. lipolytica*. **Quantitative RT-PCR analyses of ABC1, ABC2, ABC3 and ABC4 in *Y. lipolytica *cells upon treatment with octane, nonane, decane, undecane and dodecane (C8-C12). Each value of qRT-PCR was normalized to β-actin expression levels and expressed as the fold change relative to the levels detected in control samples, which were cells without alkane treatment and set equal to 1. Error bars represent the standard deviation (SD) of three biological replicates. Primers for qRT-PCR are listed in Table S1.

To assess whether ABC1, ABC2, ABC3 and/or ABC4 transporters were involved in alkane transport in *Y. lipolytica*, we analysed the effects of alkanes with different chain lengths (C8-C12) on the transcription levels of these four ABC transporter genes using quantitative RT-PCR. The reason that C8 to C12 alkanes were tested in this study was that longer alkanes showed no toxicity toward *S. cerevisiae* (data not shown). Compared with control samples without alkane treatment, the transcription levels of *ABC1* and *ABC4* showed no change when the cells were treated with different alkanes (C8-C12) (Figure 1). However, the mRNA levels of ABC2 were significantly increased when *Y. lipolytica* was treated with octane (C8), nonane (C9), decane (C10) and undecane (C11) (p<0.05), while the mRNA levels of ABC3 were significantly increased toward nonane (C9) and decane (C10) (p<0.05) (Figure 1). These results strongly suggested that two of the ABC transporters, ABC2 and ABC3, might play a critical role in the transport of alkanes for C8, C9, C10 and C11 alkanes. Thus, based on the qRT-PCR results, ABC2 and ABC3 were chosen for further analysis of their alkane transport capability.

### Expression and subcellular localization of ABC2 and ABC3

**Figure 2:**
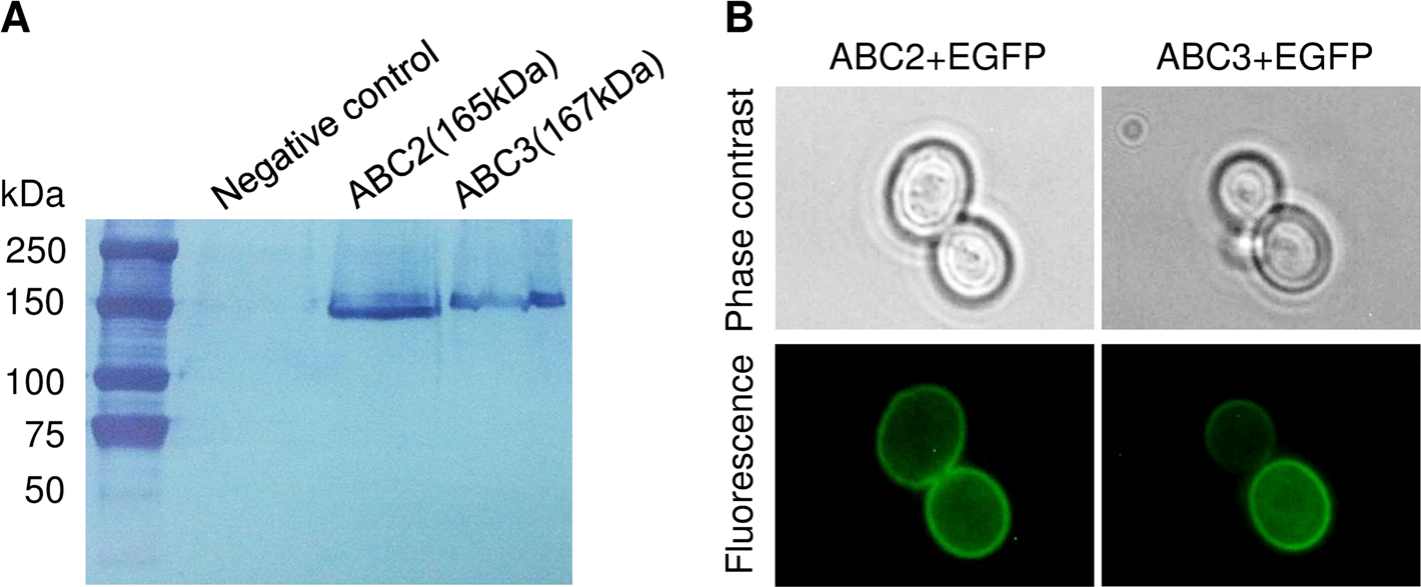
**Expression and subcellular localization of ABC2 and ABC3. (A) **Expression of ABC2 and ABC3. The expression of ABC2 and ABC3 carrying 6xHis tag was confirmed by western blot analysis. The positions of molecular mass markers are indicated at left. Western blot analyses were performed as described under “Methods”. **(B) **Subcellular localization of ABC2 and ABC3 determined by fluorescence microscopy. Yeast cells carrying plasmids encoding ABC2-EGFP or ABC3-EGFP fusion proteins were cultured and harvested. Phase contrast figures and fluorescence figures are shown.

To confirm the expression of these two transporters in *S. cerevisiae*, a 6x His tag was attached to the C terminus of ABC2 and ABC3. Through immunodetection of 6x His-tagged proteins, specific bands were assigned to ABC2 (165 kDa) and ABC3 (167 kDa) (Figure 2A). This western blot result confirmed the expression of ABC2 and ABC3 in *S. cerevisiae*. Next, to further analyse the localization of ABC2 and ABC3, each of them was tagged with EGFP at its C terminus. As shown in Figure 2B, strong fluorescence was observed on the plasma membrane of cells containing ABC2-EGFP and ABC3-EGFP fusion proteins, respectively. These results suggest that ABC2 and ABC3 were expressed and located on the plasma membrane of *S. cerevisiae*.

To investigate whether EGFP would affect the localization of the transporters, the functionality of ABC2- and ABC3-EGFP was compared with that of ABC2 and ABC3. This functionality was determined by the viability of the cells in the presence of alkanes. The growth patterns of strains expressing ABC2/3-EGFP fusion protein and ABC2/3 were similar (data not shown). These data indicate that EGFP fusion proteins function similarly as the untagged proteins. Thus, EGFP tag does not affect the localization of the transporter proteins.

### ABC2 and ABC3 contribute to increasing alkane tolerance

**Figure 3:**
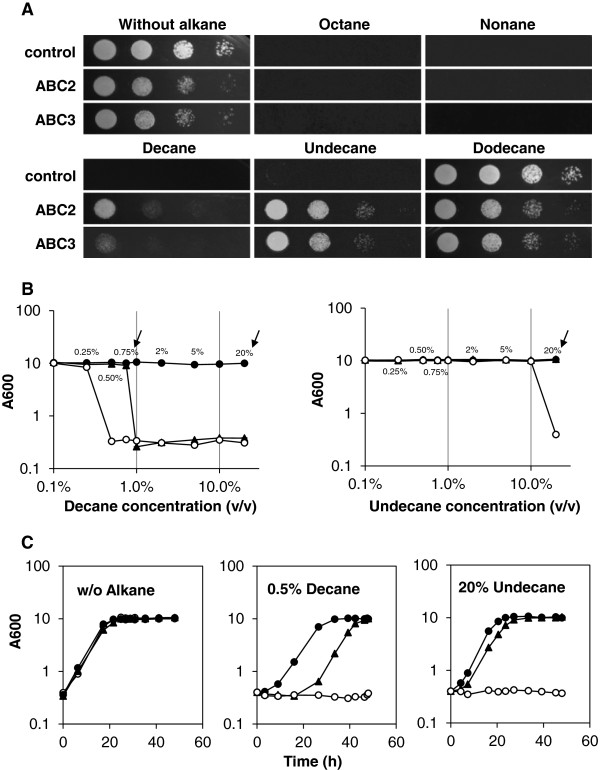
**Alkane susceptibility assay for *S. cerevisiae*. **Alkane susceptibility assays were performed in *S. cerevisiae* cells expressing ABC2, ABC3 or with an empty plasmid. **(A) **Alkane susceptibility assay on agar plates. Serial 10-fold dilutions (from left to right: non diluted, 10^-1^, 10^-2^, 10^-3^) of cells were spotted on agar plates with alkanes (octane, nonane, decane, undecane and dodecane) as vapor phase. Plates were incubated at 28°C for 2 days. **(B) **Alkane susceptibility assay in liquid culture. Overnight cell culture was diluted into induction medium (final OD_600_=0.4) with alkanes (decane or undecane). The cell culture was incubated for 48 h at 28°C. The OD_600 _value of each sample was determined and plotted against its corresponding alkane concentration (0.1%, 0.25%, 0.5%, 0.75%, 1%, 2%, 5%, 10% and 20% vol/vol). Each point represents the mean of three biological replicates; standard deviations are presented. Arrows indicate increased cell tolerance towards alkanes. **(C) **Growth file of cells in alkane-inhibiting conditions (0.5% (vol/vol) decane or 20% (vol/vol) undecane). Symbols for strains are: control sample with empty plasmid (open circle), cells expressing ABC2 (filled circle), cell expressing ABC3 (filled triangle).

After confirming that ABC2 and ABC3 were expressed on the cell membrane, we conducted susceptibility assays to study the effect of ABC2 and ABC3 on the tolerance of the cells toward alkanes. The toxic effects of alkanes on *S. cerevisiae* with ABC2 and ABC3 were measured through alkane susceptibility assays on agar plates. Figure 3A shows that octane, nonane, decane and undecane were toxic to *S. cerevisiae* cells. However, in cells expressing ABC2 and ABC3, cellular tolerance toward decane and undecane was considerably improved. It was observed that the expression of ABC2 led to higher tolerance toward decane than ABC3. Note that ABC1 and ABC4 expression led to no tolerance improvement (Additional file 1: Figure S1). Therefore, the results above suggest that ABC2 and ABC3 successfully improved alkane tolerance in *S. cerevisiae*.

To further examine quantitative effects of ABC2 and ABC3 toward decane and undecane, alkane susceptibility tests in liquid culture were conducted. As shown in Figure 3B, the tolerance limit of *S. cerevisiae* was below 0.50% and 20% for decane and undecane, respectively. With ABC2 transporter, the tolerance limit was increased to over 20% for both decane and undecane. With ABC3 transporter, the tolerance limit of *S. cerevisiae* was improved to over 0.75% and 20% for decane and undecane, respectively. This result suggests that the expression of ABC2 and ABC3 transporters significantly improved the tolerance toward decane and undecane.

To study the time-course behavior of ABC2- and ABC3-expresssing *S. cerevisiae* cells in the presence of 0.5% decane and 20% undecane, which caused no growth in the wild-type cells, the growth patterns were studied. As shown in Figure 3C, while *S. cerevisiae* cells without the transporters exhibited no growth, the cells with ABC2 and ABC3 transporters grew normally after initial growth delays. ABC3 expressing cells underwent a longer growth delay under both alkane treatments, but exhibited a slightly higher growth rate with decane treatment. Note that *S. cerevisiae* cells expressing ABC2 and ABC3 transporters exhibited similar growth rates in the absence of alkanes (Figure 3C, Additional file 1: Table S2).

### ABC2 and ABC3-mediated efflux pumping of alkanes

The results of susceptibility assay on agar plates and in liquid medium showed that both ABC2 and ABC3 transporters greatly enhanced tolerance toward decane and undecane, with ABC2 leading to stronger tolerance. We therefore hypothesized that the tolerance generated by ABC2 and ABC3 was linked to the efflux pumping of decane and undecane. Alkane efflux pumping might help to maintain an intracellular alkane concentration under a toxic threshold.

**Figure 4:**
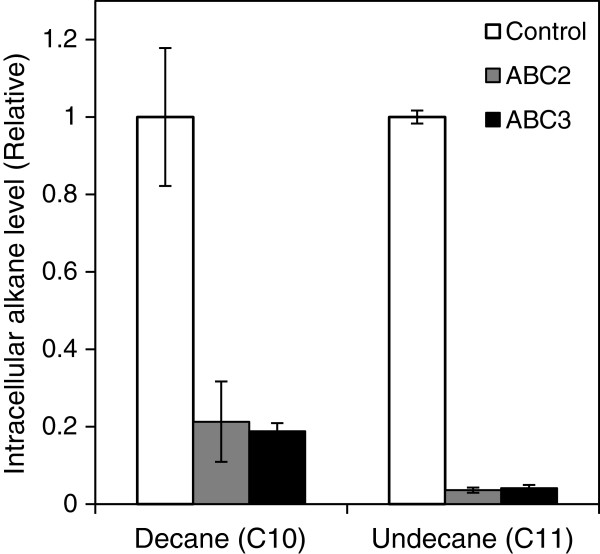
**Intracellular alkane levels of ABC2 and ABC3. *S. cerevisiae *BY4741 with and without ABC2/3 were cultured under exposure to 0.5% (vol/vol) alkane or 20% (vol/vol) undecane. **After 48 h incubation, intracellular alkane levels were measured as described under “Methods”. Intracellular alkane levels were normalized to that of control cells carrying an empty plasmid. Data shown are the mean ± SD of four biological replicates.

To verify this hypothesis, intracellular alkane accumulation was analysed after 48 h incubation with 0.5% decane or 20% undecane. As shown in Figure 4, cells expressing ABC2 and ABC3 had ~5-fold lower intracellular decane level relative to the control. Further, ABC2 and ABC3 transporters were shown to reduce the intracellular undecane level approximately 30-fold compared to the control. The sharp decrease in intracellular alkane levels strongly suggests that ABC2 and ABC3 function as decane and undecane efflux pumps.

### Glutamate is required for energy-dependent efflux pumping of ABC2 and ABC3

The main characteristic of ABC transporters is that ABC transporters utilize the energy of ATP hydrolysis to carry out biological processes. The ATP hydrolysis model is essential for ABC transporters. Thus, to better understand ABC2 and ABC3’s mechanisms in alkane export, we looked into the possible ATP hydrolysis models for both transporters. Two different models of ATP hydrolysis mechanisms were proposed for ABC transporters before: the “catalytic carboxylate” model [31] and the “catalytic dyad” model [32]. According to the “catalytic carboxylate” model, the highly conserved glutamate residue at the C terminus of the Walker B motif is essential for ATP hydrolysis. However, in the “catalytic dyad” model, interactions between glutamate of the Walker B motif and the histidine of the H-loop are a prerequisite for ATP hydrolysis.

**Figure 5:**
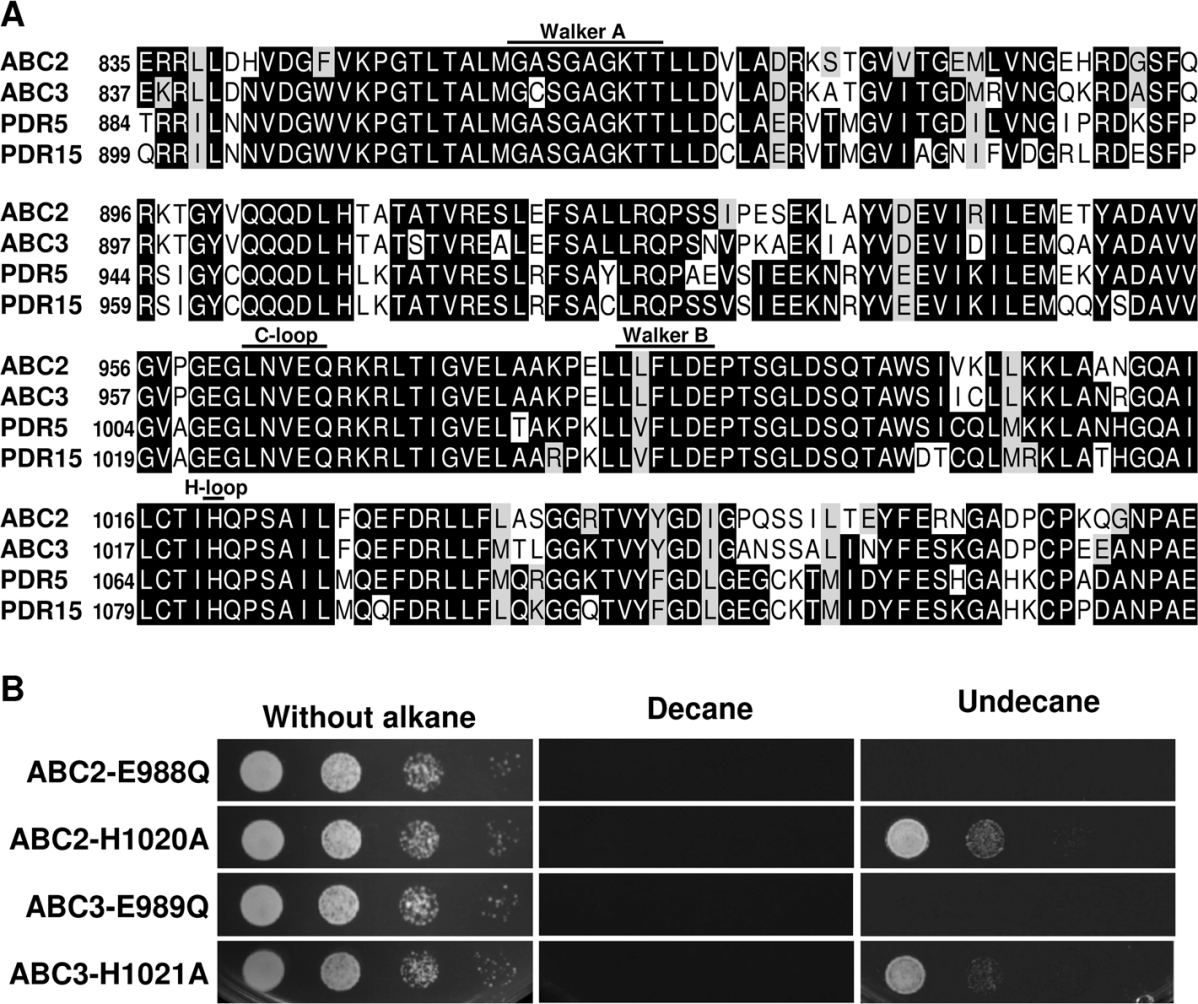
**Transporter sequence comparison and alkane susceptibility assay of ABC2 and ABC3 mutants. (A) **Multiple sequence alignment of NBD2 of ABC transporters. ABC2 [GenBank: CAG82364]; ABC3 [GenBank: CAG82646]; PDR5 [GenBank: CAA99359]; and PDR15 [GenBank: AAB64846]. Starting amino acid numbers are shown in each line. Sequences were aligned in ClustalW, and the shading was created by Mobyle Pasteur Boxshade. Single letter abbreviations for amino acid residues are as follows: A, Ala; C, Cys; D, Asp; E, Glu; F, Phe; G, Gly; H, His; I, Ile; K, Lys; L, Leu; M, Met; N, Asn; P, Pro; Q, Gln; R, Arg; S, Ser; T, Thr; V, Val; W, Trp; and Y, Tyr. **(B) **Alkane susceptibility assay with ABC2 and ABC3 mutants. Susceptibility assays were performed as described under “Methods”.

Protein analyses revealed four possible locations of characteristic domains in ABC transporters, namely two nucleotide-binding domains (NBD1 & 2) and two transmembrane domains (TMD1 & 2) [33,34]. Sequence alignment of ABC2, ABC3, PDR5 and PDR15 showed that these proteins have high similarities in NBD domains which include Walker A motif, Walker B motif, C-loop and H-loop (Figure 5A). Similar to the widely studied PDR5 model [33,34], critical amino acids such as glutamate in C-terminus of Walker B motif and histidine of H-loop are only present in NBD2 but not in NBD1 for ABC2 and ABC3 [35].

Hence, to determine the ATP hydrolysis mechanism of ABC2 and ABC3, the glutamate (E988 for ABC2 and E989 for ABC3) and the histidine (H1020 for ABC2 and H1021 for ABC3) in NBD2 of ABC2 and ABC3 were mutated to glutamine and alanine, respectively (Additional file 1: Figure S2). As shown in Figure 5B, ABC2-E988Q and ABC3-E989Q mutants were highly sensitive against both decane and undecane, while ABC2-H1020A and ABC3-H1021A mutants still showed increased tolerance against undecane. Therefore, histidine is deemed not as essential as glutamate for these transporters’ activity, and ATP is most likely to be hydrolyzed by the catalytic carboxylate mechanism.

## Discussion

In biofuel production, end products such as alkanes are frequently toxic to host cells, thereby placing a limit on the yield. Thus, cellular tolerance optimization is essential in the production of biofuels. To improve the yield and productivity, engineering strategies, including increasing tolerance toward biofuels in producing host cells, need to be developed. To this end, here, we focused on improving alkane tolerance in *S. cerevisiae*. Our alkane susceptibility assays revealed that C12 or longer alkanes exhibited little or no toxicity toward *S. cerevisiae* (data not shown), while C8-C11 alkanes were toxic. This result suggests that longer chain length alkanes have lower cell toxicity toward *S. cerevisiae*, in agreement with the previous study by Gill and Ratledge (1972) [15] demonstrating that the toxicity of alkanes is related to their chain length. The proposed mechanisms for this phenomenon are as follows. First, higher chain lengths lead to increasing molecular weight. The higher the molecular weight is, the harder it is for the compound to penetrate yeast cell membrane. Second, a previous study [18] suggested that a compound whose log *P*_*ow*_ is between 1.5 and 6.0 is considered extremely toxic to microorganisms and other living cells, such as nonane (log *P*_*ow*_ = 5.5) and decane (log *P*_*ow*_ = 6.0). Higher chain length alkanes have higher log *P*_*ow*_ value. For example, C12 alkane has log *P*_*ow*_ of 7.0 and alkanes with chain length above 12 have higher log *P*_*ow*_. This reported correlation between toxicity and log *P*_*ow*_ is in line with our observation that alkanes with longer chain length (C12 or longer) show insignificant toxicity toward *S. cerevisiae*. Since longer alkanes showed insignificant toxicity toward *S. cerevisiae,* this study was focused on improving the tolerance of *S. cerevisiae* against alkanes with C12 and below.

ABC2 and ABC3 were selected as candidates based on their potentials toward alkane efflux pumping. Although both ABC2 and ABC3 expressing cells showed increased C10 and C11 alkane tolerance, ABC2 exhibited better performance than ABC3, suggesting that ABC2 is a more effective pump for alkane transport. Thus, we looked into the possible reasons for the difference in efficiency between these two transporters. One plausible reason is the difference in kinetics of substrate–protein interactions and/or nucleotide–protein interactions between ABC2 and ABC3. For example, Low et al. (2010) [36] found that difference in TMD residues can lead to differences in ABC transporter efficiency. It was hypothesized that the TMD residues might affect substrate affinity, resulting in differences in substrate-protein kinetics. Another reason for the different efflux efficiencies may be post-translational modification patterns. Protein phosphorylation was found to be essential for the stability of the yeast multidrug transporter Pdr5p [37]. As hypothesized before, transporters, such as Pdh1p that requires more phosphorylations than Cdr1p, may take longer time to reach its active, substrate-effluxing form [38]. Thus, similar to Pdh1p, it is possible that ABC3 requires longer phosphorylation time to arrive at its active alkane-effluxing form.

Besides the difference in efflux pumping efficiencies of ABC 2 and ABC3, we also observed that ABC2 and ABC3 did not increase tolerance toward shorter chain alkanes such as C8 and C9. Future work therefore may include directed evolution of these transporters to broaden their substrate specificity, mainly toward shorter chain alkanes. For example, random mutagenesis was shown to affect substrate specificity of ABC transporters [39]. Also, protein structure studies may be needed for better understanding of the exact substrate binding and transport mechanism.

## Conclusions

In this study, we demonstrated that transporter engineering through expression of heterologous efflux pumps led to improved tolerance against alkanes in *S. cerevisiae*, a key industrial microbial host. In particular, we showed that the tolerance limit of *S. cerevisiae* was increased about 80-fold against decane. Further, we provided the evidence that the improved tolerance was primarily due to the lowering of the intracellular alkane level wherein ABC2 and ABC3 function as an efflux pump. To our knowledge, this is the first study to harness a transporter-engineering strategy for alkane tolerance improvement in eukaryotic host cells, which is readily applicable to alkane biofuel-producing microbes. We believe that our work here laid the groundwork for developing robust alkane-producing yeast cells through transporter engineering, which will greatly aid in next-generation alkane biofuel production and recovery.

## Methods

### Strains and media

All cells involved in cloning experiments were *E. coli* TOP10 (Invitrogen) unless otherwise stated. Luria-Bertani (BD) was used as the medium for cloning studies unless otherwise stated. Ampicillin (100 μg/ml) was added to the culture media for antibiotic selection where appropriate.

The yeast strains *S. cerevisiae* BY4741 (ATCC 201388) and *Y. lipolytica* CLIB122 (CIRM) were used for function characterization. *S. cerevisiae* BY4741 was cultured in rich medium (YPD), synthetic minimal medium lacking uracil (SC-U) or induction medium. YPD medium (1% yeast extract, 2% peptone and 2% D-glucose) was used to routinely maintain wild type strain. SC-U medium (0.67% yeast nitrogen base, 0.192% uracil dropout and 2% raffinose) was used for growing pYES2 transformants. Induction medium (0.67% yeast nitrogen base, 0.192% uracil dropout, 1% raffinose and 2% galactose) was used for protein induction in *S. cerevisiae* cells. Medium containing 0.67% yeast nitrogen base supplemented with 0.5% casein hydrolysate and 2% glucose was used for growth of *Y. lipolytica* for qRT-PCR sample preparation. Solid media were supplemented with 2% agar. Yeast growth media components were purchased from Sigma-Aldrich.

Alkanes (octane (C8), nonane (C9), decane (C10), undecane (C11) and dodecane (C12)) purchased from Sigma-Aldrich were added to culture media for protein function analysis where appropriate.

### Plasmid construction

A list of the oligonucleotides used is shown in Additional file 1: Table S1. Plasmid pYES2 (Invitrogen) with the GAL1 promoter was used as an expression vector.

To clone 6x His-tagged ABC2, genomic DNA of *Y. lipolytica* CLIB122 was used as a PCR template with two pairs of primers ABC2-F1/ABC2-R1, and ABC2-F2/ABC2-R2. The two PCR products were combined through the Splicing Overlap Extension (SOE) method [40] using flanking primers ABC2-F1 and ABC2-R2. The resulting DNA fragment was digested with HindIII and NotI and cloned into pYES2 cut with the same restriction enzymes, creating pYES2ABC2. Plasmid pYES2ABC3 was constructed as for pYES2ABC2. Site-directed mutagenesis of the transporters ABC2-E988Q, ABC2-H1020A, ABC3-E989Q and ABC3-H1021A were constructed by mutating glutamate to glutamine and histidine to alanine respectively.

Plasmid pYES2ABC2-EGFP, which encodes yeast enhanced green fluorescent protein (EGFP) at the C-terminus of the ABC2 open reading frame, was constructed as follows. We used pYES2ABC2 as a PCR template with primer set ABC2-F1 and ABC2-EGFP-R2. The resulting DNA fragment was digested with HindIII and NotI and cloned into pYES2 cut with the same restriction enzymes, creating pYES2ABC2-1. EGFP was amplified from pKT127 (Euroscarf) [41] using primer set EGFP-F and EGFP-R, digested with NotI and SphI and inserted into the same restriction sites of pYES2ABC2-1 to create pYES2ABC2-EGFP. Plasmid pYES2ABC3-EGFP was constructed as for pYES2ABC2-EGFP. For construction of pYES2EGFP, EGFP was amplified by PCR from pKT127 using primers EGFP-control-F and EGFP-R, digested with NotI and SphI and cloned into pYES2. All restriction and ligation enzymes were purchased from New England Biolabs (NEB).

### Quantitative RT-PCR

Total RNA samples from *Y. lipolytica* CLIB122 cells, which were treated and untreated with alkane for 24 h, were prepared using RNeasy Mini Kit (Qiagen), followed by cDNA synthesis using H minus Reverse transcriptase kit (Fermentas). Quantitative RT-PCR analysis was performed on the Bio-Rad iQ5 real-time PCR detection system using SsoFast EvaGreen Supermix kit (Bio-Rad). The actin gene (YALI0D08272g) [42] was used as reference gene for *Y. lipolytica*. Relative mRNA levels were derived using comparative C_T_ method. PCR primer sequences are listed in Additional file 1: Table S1.

### Western blot analysis

*S. cerevisiae* cells carrying the plasmids encoding the 6x His-tagged ABC2 and ABC3 were cultured in induction medium and harvested at OD_600_=1-2 (early exponential phase). The protein extraction method here is based on alkaline lysis [43] and glass bead lysis [44] methods. The following handling process was carried out in the cold room (~4°C). Cell pellets (around 14 mg) were re-suspended in 300 μl cold lysis buffer (0.1 M NaOH, 2% β-mercaptoethanol, and protease inhibitor mixture (Roche Applied Science)). After 5 min, glass beads (425-600 μm, Sigma) were added to the suspension. The cells were lysed by vortexing for 2 min. The lysate obtained was clarified by transferring supernatant into a new tube. Proteins in the lysate were fully dissolved by adding SDS (final concentration around 2%) and gently stirring for 10 min. After centrifugation, the supernatant was mixed equally with Laemmli sample buffer (Bio-Rad) and separated on a SDS-polyacrylamide gel. The sample gels were used for blotting. Proteins were blotted onto a 0.2 μm nitrocellulose membrane (Bio-Rad) through Trans-Blot Turbo Blotting System (Bio-Rad). 6x His-tagged ABC2 and ABC3 were detected using anti-6x His-tag antibody (HRP) (ab1187, Abcam) and 3,3^′^,5,5^′^-Tetramethylbenzidine (TMB) liquid substrate system (Sigma).

### Fluorescence microscopy

*S. cerevisiae* BY4741 cells carrying plasmid pYES2ABC2-EGFP or pYES2ABC3-EGFP were grown to the early exponential phase in induction medium, harvested and mounted on the poly-L-lysine-coated slide glass. EGFP fluorescence was analysed with a fluorescent microscope (Zeiss Axio Scope A1).

### Alkane susceptibility assays

*Alkane susceptibility test on agar plate:* Alkane susceptibility test on plates was performed according to the methods of Mauersberger et al. [28,29]. Exponentially growing cells in induction medium were centrifuged and re-suspended until OD_600_ reaches 1. Ten microliter aliquots of successive 10-fold dilutions (non diluted, 10^-1^, 10^-2^, 10^-3^) of the cells were spotted onto the induction medium plate. Alkanes were supplied as vapour phase by placing 200 μl alkane on a sterile filter paper in the lid of the petri dish. Plates were incubated at 28°C for 2 days.

*Alkane susceptibility test in liquid culture:* Overnight cultures were diluted into 5 ml induction medium in 50 ml glass bottle (Sigma) at an initial OD_600_ of 0.4. Alkanes were added at different final concentrations. Bottles were sealed tightly with butyl rubber stopper (Sigma) and silver aluminum seal (Sigma). Liquid culture was performed at 28°C with shaking. Growth was monitored by measuring the OD_600_ at different time points. Cell cultures used for time point OD_600_ checking were collected from the glass bottle using needles and syringes.

### Determination of intracellular alkane levels

After induction for 48 h with or without addition of alkanes, *S. cerevisiae* cells transformed with pYES2, pYES2ABC2 and pYES2ABC3 were harvested at 6000 g for 5 min at 4°C. After washing with 50 mM Tris.Cl, the cells were equally divided into two parts, one part for intracellular alkane extraction and the other for determination of total protein content. For alkane extraction, cell pellets were re-suspended in freshly prepared HPLC grade Chloroform/Methanol (v/v, 2:1). Dodecane was added into cell suspension as an internal standard. Acid-washed glass beads were added until the suspension was covered. Cells were then lysed by mechanical agitation using FastPrep-24 (MPBio) for 6 min at 6 m/s. The crude extract was obtained by pipettes. After addition of autoclaved ddH_2_O, the crude extract was emulsified for 10 min by inversion. After centrifugation, the crude extract was separated into two phases. The bottom phase containing alkane was transferred into a new 1.5 ml microcentrifuge tube and purified as above with chloroform and ddH2O until particulate matter was no longer observed. The purified solution was transferred into a clear GC vial for GC analysis. To check the total protein content, the cell pellets were re-suspended into 50 mM Tris.Cl and lysed via mechanical agitation with acid-washed glass beads. Protein content of obtained crude extract was determined using the Bradford protein assay (Bio-Rad). Intracellular alkane levels were normalized to internal standard and cell lysate protein content [45,46].

## Competing interests

The authors declared competing financial interests.

## Authors’ contribution

BC and MWC conceived the project, designed the experiments, and wrote the manuscript. BC and HL conducted the experiments and analyzed the data. MWC supervised the project. All authors read and approved the final manuscript.

## Supplementary Material

Additional file 1: Table S1 Primers used in this study. Restriction sites are bold. **Table S2. **Specific growth rates under C10 and C11 alkane treatments. **Figure S1. **Alkane susceptibility assay with ABC1 and ABC4. Serial 10-fold dilutions (from left to right: non diluted, 10-1, 10-2, 10-3) of cells were spotted on agar plates with alkanes (octane, nonane, decane, undecane and dodecane) as vapor phase. **Figure S2.** To gengerate a topology model of ABC2 (A) and ABC3 (B), the TMHMMfix algorithm was used (http://www.sbc.su.se/~melen/TMHMMfix/). the resulting model was visualized by the TOPO2 transmembrane protein display software (http://www.sacs.ucsf.edu/cgi-bin/open-topo2.py). The Walker A motifs are shown in blue, the Walker B motifs are shown in green, and the C-loop are shown in orange. Residues that were mutated in this study are shown in red. Click here for file
