# Supplementary material for: Transporter engineering for improved tolerance against alkane biofuels in Saccharomyces cerevisiae
Source: Biotechnol Biofuels. 2013 Feb 13;6:21. doi: 10.1186/1754-6834-6-21 (PMC3598725; doi:10.1186/1754-6834-6-21)

Table S1. Primers used in this study. Restriction sites are bold.

Primer name	Primer sequence from 5' to 3'
ABC2-F1	ACCC**AAGCTT**TAAAAAATGTCTCAAACACCTCCAGACTACACTGGTCTGG
ABC2-R1	CATCTCGGGGGACAC/CCTGATAATTCCATT
ABC2-F2	AATGGAATTATCAGG/GTGTCCCCCGAGATG
ABC2-R2	ATAAGAAT**GCGGCCGC**CTAATGGTGATGGTGATGATGAGCCTTCTGAGCCTTGGA
ABC3-F1	ACGG**GGTACC**TAAAAAATGTCTGATCCTGTACCGATCACGCAGG
ABC3-R1	GATTTTTCGGGGAATGC/CCTGATAATTCCATTCA
ABC3-F2	TGAATGGAATTATCAGG/GCATTCCCCGAAAAATC
ABC3-R2	ACAT**GCATGC**CTAATGGTGATGGTGATGATGGGCCTTCTGCTTTCGAGACTTTTTG
ABC2-E988Q-R1	GACCGGAAGTAGGTTGATCCAGGAACAGC
ABC2-E988Q-F2	GCTGTTCCTGGATCAACCTACTTCCGGTC
ABC2-H1020A-R1	GCAGAGGGCTGAGCAATGGTACACAGAATAGC
ABC2-H1020A-F2	GCTATTCTGTGTACCATTGCTCAGCCCTCTGC
ABC3-E989Q-R1	GGAAGTGGGTTGATCAAGGAACAGC
ABC3-E989Q-F2	GCTGTTCCTTGATCAACCCACTTCC
ABC3-H1021A-R1	GCAGAGGGCTGAGCAATAGTACACAG
ABC3-H1021A-F2	CTGTGTACTATTGCTCAGCCCTCTGC
ABC2-EGFP-R2	ATAAGAAT**GCGGCCGC**AGCCTTCTGAGCCTTGGACTTCTTG
ABC3-EGFP-R2	ACAT**GCATGC**AACATGGAT**GCGGCCGC**GGCCTTCTGCTTTCGAGACTTTTTGG
EGFP-F	ATAAGAAT**GCGGCCGC**GATCGGTGACGGTGCTGGTTTAA
EGFP-R	ACAT**GCATGC**TTAATGGTGATGGTGATGATGTTTGTACAATTCATCCATACCATGG
EGFP-control-F	ATAAGAAT**GCGGCCGC**TAAAAAATGTCTAAAGGTGAAGAATTATTCACTGGTGTTG
ABC1-qPCR-F	CGACGACAACAGCGTGAGGAACT
ABC1-qPCR-R	CCACTGAGACACGGCGAAATCAGT
ABC2-qPCR-F	CCTGTTGTGTGGGCTGAGTCTCCT
ABC2-qPCR-R	TCGCTCGGTAGTGTTCGTCTGCAC
ABC3-qPCR-F	CGTGGCAGGCTTTCATGGGCT
ABC3-qPCR-R	AAGAGGGCTCCTCGTTCGCCA
ABC4-qPCR-F	GGCCACCAAAGAGCAGATGCCTG
ABC4-qPCR-R	ACGTTACCGGGGCTCGTCCAA
Actin-F	TCCAGGCCGTCCTCTCCC
Actin-R	GGCCAGCCATATCGAGTCGCA

Table S2. Specific growth rates under C10 and C11 alkane treatments

		$\mu(h^{-1})$
w/o alkane treatment	empty plasmid	0.189±0.008
	ABC2 expressing	0.173±0.019
	ABC3 expressing	0.164±0.011
with 0.5% C10 treatment	empty plasmid	0
	ABC2 expressing	0.143±0.002
	ABC3 expressing	0.168±0.009
with 20% C11 treatment	empty plasmid	0
	ABC2 expressing	0.204±0.021
	ABC3 expressing	0.178±0.015

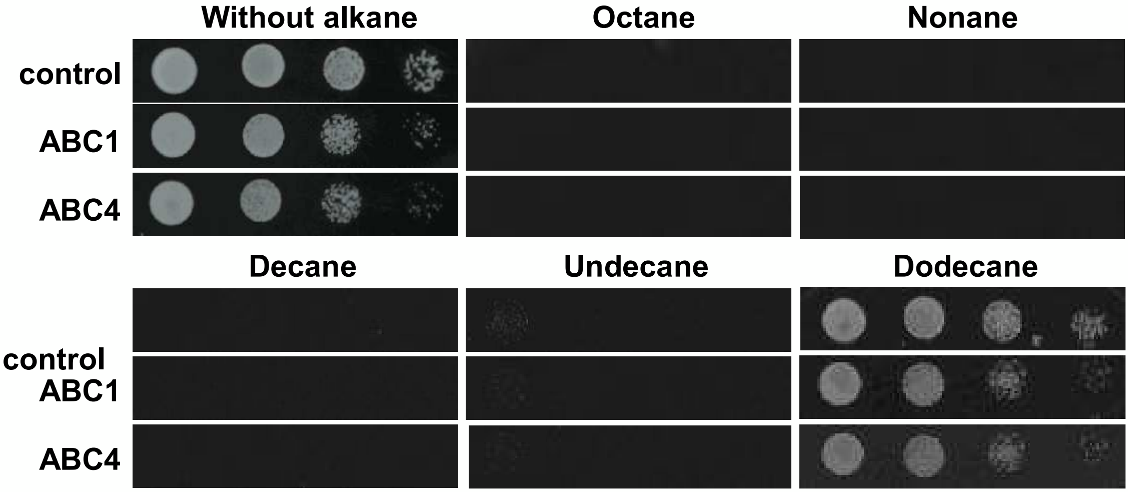

Figure S1. Alkane susceptibility assay on agar plates. Serial 10-fold dilutions (from left to right: non diluted, 10^{-1} , 10^{-2} , 10^{-3}) of cells were spotted on agar plates with alkanes (octane, nonane, decane, undecane and dodecane) as vapor phase.

Figure S2. Topology model of ABC2 and ABC3. To generate a topology model of ABC2 (A) and ABC3 (B), the TMHMMfix algorithm was used (www.sbc.su.se/melen/TMHMMfix). The resulting model was visualized by the TOPO2 transmembrane protein display software (www.sacs.ecsf.edi/TOPO2). The Walker A motifs are shown in blue, the Walker B motifs are shown in green, and the C-loop are shown in orange. Residues that were mutated in this study are shown in red.

A

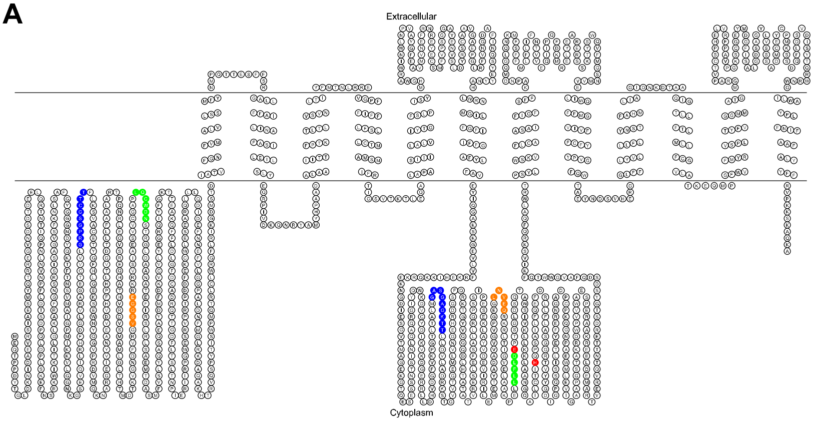

B

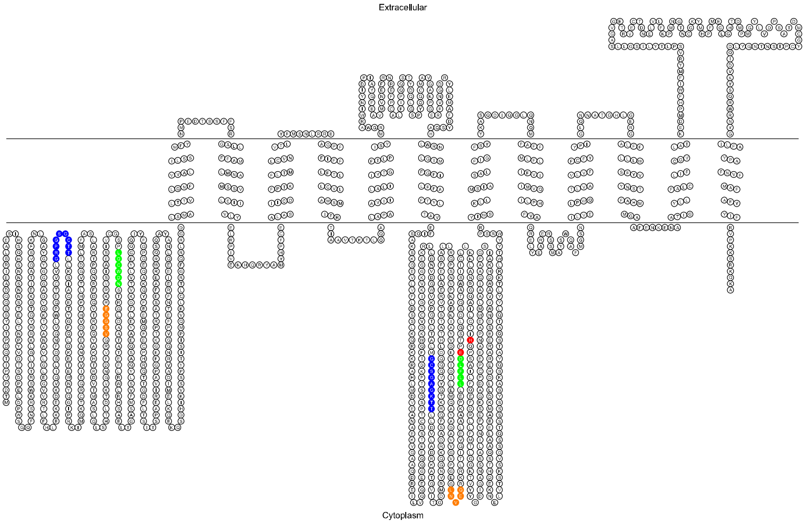

Supplement: Additional file 1: Table S1 — Primers used in this study. Restriction sites are bold. Table S2. Specific growth rates under C10 and C11 alkane treatments. Figure S1. Alkane susceptibility assay with ABC1 and ABC4. Serial 10-fold dilutions (from left to right: non diluted, 10-1, 10-2, 10-3) of cells were spotted on agar plates with alkanes (octane, nonane, decane, undecane and dodecane) as vapor phase. Figure S2. To gengerate a topology model of ABC2 (A) and ABC3 (B), the TMHMMfix algorithm was used (http://www.sbc.su.se/~melen/TMHMMfix/). the resulting model was visualized by the TOPO2 transmembrane protein display software (http://www.sacs.ucsf.edu/cgi-bin/open-topo2.py). The Walker A motifs are shown in blue, the Walker B motifs are shown in green, and the C-loop are shown in orange. Residues that were mutated in this study are shown in red. [file 1754-6834-6-21-S1.pdf]
